# Cholesterols, Apolipoproteins, and Their Associations with the Presence and Severity of Diabetic Retinopathy: A Systematic Review

**DOI:** 10.3390/vision6040077

**Published:** 2022-12-16

**Authors:** Soefiandi Soedarman, King Hans Kurnia, Alberthus Donni Budi Prasetya, Muhammad Bayu Sasongko

**Affiliations:** 1 Department of Ophthalmology, Faculty of Medicine, Public Health and Nursing, Universitas Gadjah Mada, Yogyakarta 55281, Indonesia; 2 JEC Eye Hospitals & Clinics, Jakarta 10310, Indonesia; 3 Sardjito Eye Center, Dr. Sardjito General Hospital, Yogyakarta 55281, Indonesia

**Keywords:** apolipoprotein, cholesterol, diabetic retinopathy (DR), diabetes mellitus, systematic review

## Abstract

Serum apolipoproteins have been reported as a more significant marker for diabetic retinopathy (DR) compared with serum cholesterols. This article aims to review the associations between serum cholesterols and apolipoproteins, and the presence and severity of DR. The protocol of this systematic review was registered at the PROSPERO registry (CRD42022303331). We conducted a systematic search of literature published between 2011 to 2022 using the search terms “serum cholesterol” AND/OR “lipoprotein” AND/OR “apolipoprotein” AND/OR “diabetic retinopathy”. Fifteen studies were included in this review. Six studies assessed the association between serum cholesterols, apolipoproteins, and the presence of DR. Three studies reported lower levels of apolipoprotein A1, and one study reported higher levels of apolipoprotein B in patients with DR. The remaining nine studies compared serum cholesterol and apolipoprotein levels according to DR severity. Patients with more severe grades of DR presented with lower apolipoprotein A1 in six (66.7%) studies, higher apolipoprotein B levels in seven (77.8%) studies, and a higher apolipoprotein B/apolipoprotein A1 ratio in six out of seven (85%) studies. In conclusion, serum apolipoproteins, in particular the apolipoprotein B/apolipoprotein A1 ratio, were a more consistent marker for DR severity compared with serum cholesterols.

## 1. Introduction

Diabetic retinopathy (DR) is a leading cause of vision loss globally, affecting approximately 1 in every 4 individuals with type 2 diabetes mellitus (DM) [1]. Progression of DR has been mainly associated with diabetes duration and glycemic control [2]; therefore, markers of glycemic control, such as blood glucose levels and HbA1C, have been conventionally used as predictors of disease progression and treatment outcomes [3,4]. However, risk factors for DR and its progression are multifactorial. Other risk factors for DR have been elucidated, including hypertension, dyslipidemia, obesity, puberty, and pregnancy [5].

Previous studies have reported the significance of serum cholesterols and their association with DR [6,7]. Dyslipidemia has been reported as one modifiable risk factor for DR in large studies [8]. The presence of dyslipidemia may partly explain why certain individuals demonstrated constant progression of DR despite good glycemic control [9,10,11]. However, results of studies regarding the association between conventional cholesterol parameters and DR were inconsistent [8]. Furthermore, results from clinical trials suggest that the beneficial effects of cholesterol reduction on DR progression may also be attributed to other mechanisms, in addition to direct control of dyslipidemia. These mechanisms include upregulation of apolipoprotein A1 and antioxidant enzymes [12].

In recent years, several studies have investigated the association between serum apolipoproteins and DR. Further to conventional cholesterols, apolipoproteins take into account the modification and size distributions of the constituting lipid particles; therefore may provide a finer depiction of the complex lipid metabolism system [8]. Some studies have shown that serum apolipoproteins were significantly associated with the presence of DR and improved the prediction model for DR in diabetic patients [7,13,14,15,16]. Consequently, the role of apolipoproteins on DR and its progression is promising for exploration. However, the small sample size of existing studies was a major limitation in drawing a strong conclusion. 

Therefore, we aimed to systematically review all published studies that report the associations between serum cholesterols, apolipoproteins, and DR; then, summarize the association between serum cholesterol and apolipoprotein parameters and the presence and severity of DR in diabetic patients. 

## 2. Materials and Methods

### 2.1. Search Strategy

**Figure 1:**
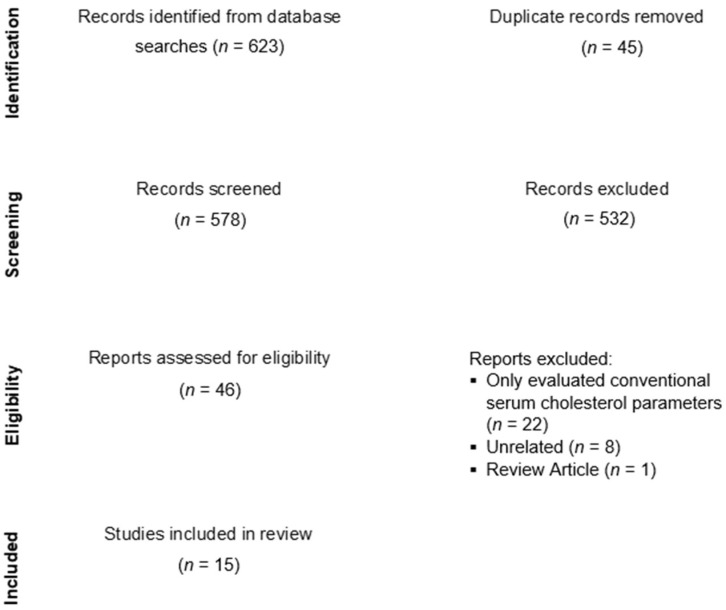
PRISMA flow chart of study selection.

The protocol of this systematic review was registered at the PROSPERO registry (https://www.crd.york.ac.uk/prospero/), accessed on 15 February 2022, with the protocol number CRD42022303331. We conducted a systematic literature search for studies assessing the associations of serum cholesterol and apolipoprotein parameters with the presence and severity of DR. We searched all studies published in English available from Pubmed, Scopus, Web of Science, EMBASE, and Cochrane Library databases in the last 11 years (2011 to 2022) using the combinations of the following terms: “serum cholesterol” AND/OR “lipoprotein” AND/OR, “apolipoprotein” AND/OR “diabetic retinopathy”. Additionally, manual searches from the reference list of major articles were also performed. Two investigators (KH and AD) independently reviewed the abstracts and full texts of the studies. Disagreement in the review process was resolved through discussion, involving the third investigator (SS). The study-selection process was reported according to the Preferred Reporting Items for Systematic Review and Meta-Analyses (PRISMA) guidelines (Figure 1). 

### 2.2. Eligibility Criteria

Studies were selected for review according to the following inclusion criteria: (1) population-based or hospital-based studies with cross sectional, case control, and cohort designs, and (2) studies in adults with type 2 DM. We excluded: (1) studies which only involve patients with type 1 DM or gestational DM, and (2) studies evaluating only conventional cholesterol parameters (triglyceride, high-density lipoprotein (HDL) cholesterol, low-density lipoprotein (LDL) cholesterol, and total cholesterol).

### 2.3. Data Extraction and Quality Assessment

Data extracted from the studies included: author, year, study design, patients’ age, diabetes duration, HbA1C level, use of lipid-lowering treatment, apolipoprotein measurement assay, number of study participants, classification of DR severity, levels of apolipoprotein parameters (Apo-A1, Apo-B, Apo-B/Apo-A1 ratio, and other available lipoprotein parameters), levels of cholesterol parameters (triglyceride, HDL cholesterol, and LDL cholesterol), and measures of effect or associations with DR outcomes (*p* value, odds/hazard ratio, and 95% confidence interval). Data were extracted independently by two investigators (KH and AD) and recorded in a dedicated spreadsheet. Quality assessment of the studies was performed by appraising the studies using the Strengthening the Reporting of Observational Studies in Epidemiology (STROBE) checklist. The spreadsheet containing the extracted data was checked for errors and discrepancies by the third investigator (SS). Any disagreement was resolved by discussion.

## 3. Results

Literature search yielded a total of 578 articles after removal of duplicates, and 46 studies passed the article screening process. Thirty-one studies were excluded (22 studies only evaluated conventional cholesterol parameters, eight studies were unrelated to the review, and there was one review article), with the remaining 15 studies relevant to the clinical question were reviewed (Figure 1). Characteristics of the reviewed studies are presented in Table 1. All were observational studies. In a majority of the studies, patients with DR, especially more severe grades of DR, were more likely to have experienced a longer duration of DM and higher levels of baseline HbA1C. Studies included in this review did not restrict the use of lipid-lowering drugs among study patients. In all studies that reported the use of lipid-lowering drugs, there were no significant differences in the proportion of lipid-lowering drug use among groups of patients.

### 3.1. Association between Serum Cholesterol, Apolipoprotein Parameters, and the Presence of DR

Six studies evaluated the association between cholesterol and apolipoprotein parameters and the presence of DR (Table 2). In four studies, there were no significant differences on any cholesterol parameters between patients with or without DR [14,21,25,29]. However, Chung et al. [26] reported a significantly lower level of HDL in patients with DR (1.12 ± 0.37 mmol/L) compared with patients without DR (1.17 ± 0.35 mmol/L) and Malaguarnera et al. [19] reported a significantly higher level of LDL in patients with DR compared with patients without DR (4.19 ± 0.99 mmol/L and 3.87 ± 0.95 mmol/L, respectively; *p* = 0.049). Other cholesterol parameters in the two studies were similar between both groups.

Conversely, apolipoprotein parameters showed significant differences between patients with and without DR. In particular, Apo-A1 showed significant difference between both groups in five studies, although the findings were contradictory. Aryan et al. [21] and Malaguarnera et al. [19] reported that for patients with microvascular complications of type 2 DM and DR, respectively, Apo-A1 levels were significantly higher, while the remaining three studies observed that patients without DR have significantly higher levels of Apo-A1 [14,25,26]. Among five studies that assessed the association between Apo-B and the presence of DR, Apo-B levels were not significantly different among patients with and without DR in four studies [19,21,26,29]. Meanwhile Sasongko et al. [14] observed that Apo-B level was significantly higher in patients with DR, and an increased Apo-B/Apo-A1 ratio was associated with increased DR incidence. Zhang et al. [25] reported that Apo-CIII and Apo-E levels, and their ratio to Apo-A1 were significantly higher in DR patients. Levels of Apo-CIII of ≥6.3 μmol/L, Apo E of ≥1.1 μmol/L, Apo-CIII/Apo-A1 ratio of ≥ 0.9, and Apo-E/Apo-A1 ratio of ≥0.2 were associated with increased DR risk. Three studies assessed the association of Lp(a) with the presence of any DR, and in two studies Lp(a) was positively associated with DR [19,29].

In the hazard and regression analysis performed in five studies, higher level of Apo-A1 was a significant protective factor against the presence of any type of DR [14,25,26]. Sasongko et al. [14] and Liu et al. [29] reported that an increased level of Apo-B was a risk factor for the presence of DR, while other two studies found no significant role of Apo-B on the presence of any type of DR [21,26]. For other apolipoproteins, Zhang et al. [25] found increased levels of Apo-CIII, Apo-E, and their ratios to Apo-A1, were risk factors for the presence of any DR, while Aryan et al. [21] and Liu et al. [29] demonstrated that increased Lp(a) was not a significant risk factor for the presence of any DR.

### 3.2. Association between Serum Cholesterol, Apolipoprotein Parameters, and DR Severity

Nine studies evaluated the association between serum cholesterol and apolipoprotein parameters and DR severity. The summary of the studies is presented in Table 3. In seven studies that reported the results of conventional cholesterol parameters, patients with proliferative diabetic retinopathy (PDR) or vision-threatening diabetic retinopathy (VTDR) had significantly lower levels of HDL in four studies [17,18,20,24], higher levels of triglyceride in three studies [20,22,23], and higher levels of LDL in one study [22], when compared with patients with non-proliferative diabetic retinopathy (NPDR) or no DR. There was a significant inverse association between HDL cholesterol levels and DR severity (*p* = 0.02) [20]. HDL/LDL ratio was also significantly lower in patients with PDR compared with patients with mild NPDR [18].

In contrast, more consistent findings are observed for the comparison of apolipoprotein levels between patients with PDR or VTDR and patients with NPDR or no DR. Patients with PDR or VTDR demonstrated lower Apo-A1 levels in six studies [17,18,20,22,23,24], and higher Apo-B levels in seven studies [13,17,20,22,23,24,27]. Further logistic regression analysis revealed that lower Apo-A1 levels [18] and higher Apo-B levels [13,27] were significant risk factors for the progression of DR severity.

Among the studies, the unit of measurement for Apo-A1 and Apo-B levels varied. This variation was related to the devices and methods used by the laboratories. Therefore, using the Apo-B/Apo-A1 ratio could avoid the effect of these variations. Among eight studies that evaluated the Apo-B/Apo-A1 ratio, six studies [13,17,18,20,22,24] found significant difference in the Apo-B/Apo-A1 ratio between patients with PDR or VTDR and patients with NPDR or no DR. Patients with PDR or VTDR showed increased Apo-B/Apo-A1 ratio compared with patients with NPDR or no DR. Logistic regression analysis performed in four studies [13,17,18,27] showed that increased Apo-B/Apo-A1 ratio was a significant risk factor for PDR or VTDR. Moosaie et al. [27] did not observe a significant difference in the Apo-B/Apo-A1 ratio between both groups, but logistic regression analysis demonstrated that increased Apo-B/Apo-A1 ratio was a risk factor for more severe DR types (OR 1.07; 95% CI 1.03–1.1, *p* <0.01). Therefore, the Apo-B/Apo-A1 ratio was a more consistent predictor for DR severity compared with Apo-B or Apo-A1, individually.

With regards to other apolipoprotein parameters, Hu et al. [18] showed no significant difference on Apo-E levels between patients with PDR and mild NPDR (43.57 ± 13.10 g/L vs. 43.01 ± 11.12 g/L, respectively). Similarly, Moosaie et al. [27] demonstrated no significant difference between Lp(a) level in patients with PDR and NPDR (63.81 ± 35.97 mg/dL vs. 65.8 ± 28.0 mg/dL, respectively). Logistic regression analysis also showed that Lp(a) was not a significant risk factor for the progression of DR severity (OR 1.00; 95% CI 0.1–1.01; *p* = 0.29).

## 4. Discussion

Risk factors for DR are multifactorial, including diabetes duration, hypertension, hyperglycemia, dyslipidemia, obesity, puberty, and pregnancy [5,19]. Dyslipidemia, along with hyperglycemia, hypertension, and insulin resistance, alters various biochemical pathways and growth factor signaling, exert production of free radicals and reactive oxygen species, and create a pro-inflammatory cellular environment. In turn, these chains of events disrupt the blood-retinal barrier, resulting in vascular damage and neuro-glial degeneration, ultimately leading to DR progression [30,31]. Disruption of the blood-retinal barrier also causes extravasation and accumulation of lipid particles in the retinal tissue, resulting in retinal lipotoxicity and oxidative stress [12,31].

The relationship between serum cholesterol parameters and DR severity has long been studied since the Wisconsin Epidemiologic Study of Diabetic Retinopathy [32]. Results of clinical studies demonstrated that HDL was inversely correlated with the severity of DR [14,17,18,20]. In contrast, the effect of LDL and triglyceride levels on DR severity was less consistent in clinical studies [14,21,23,26]. In another study, Benarous et al. [33] observed that serum cholesterol parameters were not associated with DR, but they had significant association with clinically-significant macular edema. 

The inconsistent association between serum cholesterol parameters and DR is due to HDL and LDL levels only referring to the lipid content of the lipoproteins. HDL and LDL particles have varying amounts of cholesterol, but they contain an exact number of apolipoprotein molecules [34,35]. Therefore, measurement of conventional cholesterol parameters is affected by the modification and size distributions of the constituting lipid particles, while apolipoprotein measurement gives a more precise estimate of the true lipoprotein cholesterol levels [8,36]. Furthermore, serum cholesterol measurement are also affected by diet, pre-analytic fasting requirements, lipid-lowering drugs, and metabolic changes in diabetes [7,25]. 

There are multiple classes and subclasses of apolipoproteins. Several subclasses that have been extensively studied include Apo-A1, Apo-A2, Apo-B, Apo-CII, Apo-CIII, and Apo-E [17]. Apo-A1 is the major anti-atherogenic HDL protein component. Apo-A1 has anti-inflammatory and anti-oxidant functions against reactive oxygen species [24]. In the retina, Apo-A1 maintains the integrity of the retinal vessels and prevents DR development [18,37]. Higher Apo-A1 level in the vitreous fluid and retinal pigment epithelium also induces a protective effect against retinal lipid deposition and lipid-related inflammation [15,16,24,38]. In a study of postmortem human eyes, Simo et al. observed a higher expression of Apo-A1 mRNA and Apo-A1 levels in eyes from diabetic donors compared with eyes from nondiabetic donors. Apo-A1 mRNA expression was mainly seen in the retinal pigment epithelium (RPE), even though the eyes had not developed DR. The authors postulated that elevated Apo-A1 expression in diabetic retinal tissue serves as a protective mechanism against oxidative stress through the action of Apo-A1 in scavenging reactive oxygen species [15]. By contrast, Apo-B is the main apolipoprotein for intermediate density lipoprotein (IDL), LDL, and very-low density lipoprotein (VLDL). Apo-B is responsible for transporting lipid to the peripheral tissue, including the retina, and also exerts atherogenic effect in the blood vessels [17,24]. 

In the present review, Apo-A1 is inversely correlated with the incidence and severity of DR. Lower Apo-A1 level was found in patients with DR compared with patients without DR [14,25,26,27]. Higher Apo-A1 level was also associated with a reduced likelihood of having more severe DR [13,14,20]. Serum Apo-A1 level of ≥ 7.4 μmol/L was related to reduced risk of having any DR by 15%, and reduced risk of progression to VTDR by 35% [25]. Conversely, Apo-B showed positive correlation with DR severity in the reviewed studies [13,17,20,22]. Higher Apo-B levels were significantly associated with increased DR severity [13,17,20,22,23,24,27], but not with the presence of DR [19,21,26,27]. In a study of enucleated eyes with DR, increased expression of oxidized LDL (Apo-B100) in the retina caused apoptosis of the retinal capillary pericytes and was associated with more severe grades of DR [39].

Compared with interpreting the results of Apo-A1 and Apo-B individually, more consistent findings were found when the Apo-B/Apo-A1 ratio was analyzed [18,24,25]. Increased Apo-B/Apo-A1 ratio is associated with the presence of any DR and more severe DR. Higher Apo-B/Apo-A1 ratio was also an independent factor contributing to clinically significant macular edema and PDR in type 2 diabetic patients of over 15 years duration [13,17,18]. Apo-B/Apo-A1 ratio reflects the balance of atherogenic and anti-atherogenic factors [27,40]. The imbalance between serum levels of Apo-A1 and Apo-B was more important than the Apo-A1 level alone in the pathogenesis of PDR [7]. One study suggested that Apo-B/Apo-A1 ratio could be an important lipid biomarker in regard to the role of dyslipidemia in DR [13]. This finding can be used for assessing and initiating early treatment in DR.

Apo-CIII and Apo-E were also found in higher levels in DR patients [25]. Apo-CIII is a component of VLDL that has a pro-inflammatory and atherogenic effect [41]. On the other hand, Apo-E composes chylomicron and IDL. It has a gene polymorphic protein with three alleles, including ApoE-ε2, ApoE-ε3, and ApoE-ε4 [42]. ApoE-ε2 and apoE-ε4 have atherogenic effect, and in the retina ApoE-ε2 and ApoE-ε3 are involved in neovascular tissue formation in PDR [43,44,45]. In addition to apolipoproteins, Lp(a) is also increased in type 2 DM patients and is a strong risk factor for cardiovascular disease [46,47]. Studies showed that Lp(a) was associated with higher frequency of DR, but not with DR severity [19,21,27,48,49]. Lp(a) causes vascular damage by inducing oxidative stress, inflammatory cascade through lipoprotein oxidation, and prothrombotic effects [19,50,51]. 

This review summarizes the role of the apolipoproteins and their association with DR. However, all of the reviewed studies are observational in design. It is unknown whether interventions could alter the levels of serum apolipoproteins and exert effect on DR progression. With increased understanding of the role of apolipoproteins, future research regarding the modification of apolipoprotein levels and its effect on DR progression will be an important field of study. Furthermore, the literature regarding the role of the apolipoproteins on diabetic macular edema is scarce. Because conventional cholesterol measurements are associated with the presence of diabetic macular edema, the effect of apolipoproteins on diabetic macular edema would be a topic of interest that warrants further study.

## 5. Conclusions

In conclusion, apolipoprotein parameters are associated with the presence of DR, especially with more severe grades of DR. The association between apolipoproteins and DR is more consistent than conventional cholesterol parameters. In particular, the Apo-B/Apo-A1 ratio incorporates the atherogenic and anti-atherogenic balance of the apolipoprotein parameters. Therefore, Apo-B/Apo-A1 ratio serves as a better predictor for DR and its severity, compared with Apo-B or Apo-A1 individually.

## Figures and Tables

**Table 1:** Characteristics of reviewed studies.

Author	Age, Years	Diabetes Duration, Years	HbA1C Level, %	Lipid Lowering Treatment	Apolipoprotein Measurement Assay
Deguchi et al. [17] (2011)	NPDR: 64.8 ± 7.36 PDR: 59.4 ± 10.0	NPDR: 10.4 ± 7.17 PDR: 12.5 ± 8.72	NPDR: 7.72 ± 1.62 PDR: 7.13 ± 1.04	NPDR: 29% PDR: 24%	Serum immunonephelometric
Sasongko et al. [14] (2011)	With DR: 60 (52–66) Without DR: 58 (49–66)	With DR: 18 (10–24) Without DR: 10 (6–17)	With DR: 8.0 ± 1.2 Without DR: 7.6 ± 1.7	With DR: 58.1% Without DR: 46.3%	Serum immunonephelometric
Hu et al. [18] (2012)	NPDR: 64.44 ± 7.86 PDR: 59.28 ± 7.49	Not mentioned	Not mentioned	NPDR: 80% PDR: 88%	Serum immunoturbidimetric
Malaguarnera et al. [19] (2013)	All: 66.8 ± 12.4	With DR: 13.8 Without DR: 5.4	With DR: 8.8 ± 0.6 Without DR: 7.2 ± 0.7	Not mentioned	Serum nephelometric
Crosby-Nwaobi et al. [13] (2015)	NPDR: 67.3 ± 12.9 PDR: 66.4 ± 9.9	NPDR: 18.8 ± 8.8 PDR: 13.5 ± 6.4	Not mentioned	Not mentioned	Serum immunoturbidimetric
Prakash et al. [20] (2016)	Not mentioned	Mild NPDR: 9.5 ± 0.57 Mod NPDR: 10.50 ± 2.32 VTDR: 13 ± 1.90	Mild NPDR: 8.3 ± 0.57 Moderate NPDR: 8.67 ± 0.20 VTDR: 11.48 ± 2.25	Not mentioned	Serum immunonephelometric
Aryan et al. [21] (2017)	With DR: 57.9 ± 9.7 Without DR: 57.5 ± 9.1	With DR: 9.8 ± 7.8 Without DR: 6.5 ± 6.1	With DR: 8.1 ± 1.7 Without DR: 7.6 ± 1.5	Not mentioned	Serum immunoturbidimetric
Sharma et al. [22] (2017)	NPDR: 55.5 ± 7.0 PDR: 51.9 ± 7.5	NPDR: 10.0 ± 5.2 PDR: 14.1 ± 6.9	NPDR: 7.7 ± 1.5 PDR: 7.3 ± 1.2	Not mentioned	Serum immunoturbidimetric
Mukherjee et al. [23] (2017)	Not mentioned	Not mentioned	Mild NPDR: 8.82 ± 1.64 Mod NPDR: 6.34 ± 0.89 Sev NPDR: 7.85 ± 0.97 PDR: 7.00 ± 1.15	Not mentioned	Serum nephelometric
Ankit et al. [24] (2017)	Not mentioned	Not mentioned	Not mentioned	Not mentioned	Serum immunoturbidimetric
Zhang et al. [25] (2018)	With DR: 57.8 ± 5.8 Without DR: 56.7 ± 5.8	With DR: 8.0 ± 3.3 Without DR: 3.4 ± 1.0	With DR: 8.6 ± 1.7 Without DR: 7.9 ± 1.7	With DR: 37.2% Without DR: 35.9%	Serum immunonephelometric
Chung et al. [26] (2019)	With DR: 63 ± 11.3 Without DR: 57.8 ± 14.1	With DR: 11.3 (10.3–12.4) Without DR: 4.1 (3.8–4.4)	With DR:9.0 ± 2.2 Without DR: 8.7 ± 2.5	With DR: 36.7% Without DR: 31.9%	Serum immunoturbidimetric
Moosaie et al. [27] (2020)	NPDR: 58.92 ± 9.46 PDR: 60.9 ± 7.4	NPDR: 13.65 ± 6.41 PDR: 17.3 ± 7.9	NPDR: 7.85 ± 1.73 PDR: 8.4 ± 1.6	NPDR: 49.5% PDR: 50.5%	Serum immunoturbidimetric
Hanifah et al. [28] (2021)	With DR: 54.5 ± 6.72 Without DR: 57.4 ± 7.5	With DR: 9.8 ± 5.7 Without DR: 8.1 ± 5.5	With DR: 8.9 ± 2.5 Without DR: 8.7 ± 2.2	Not mentioned	Not mentioned
Liu et al. [29] (2022)	With DR: 56.6 ± 11.8 Without DR: 57.3 ± 10.6	With DR: 12.85 ± 8.16 Without DR: 5.24 ± 6.12	With DR: 7.70 ± 1.53 Without DR: 7.10 ± 1.29	With DR: 6.47% Without DR: 4.84 %	Serum immunoturbidimetric

Abbreviations: DR: diabetic retinopathy; NPDR: non-proliferative diabetic retinopathy; PDR: proliferative diabetic retinopathy; VTDR: vision threatening diabetic retinopathy.

**Table 2:** Summary of studies on the association of apolipoproteins and cholesterol parameters with the presence of diabetic retinopathy (DR).

Author	Number of Participants	Mean Levels of Apolipoprotein Parameters	Mean Levels of Cholesterol Parameters	Outcomes Related to the Presence of DR
Sasongko et al. [14] (2011)	With DR: 129 Without DR: 95	**Apo-A1** With DR: 1.4 ± 0.3 g/L Without DR: 1.5 ± 0.2 g/L **Apo-B** With DR: 0.9 ± 0.3 g/L Without DR: 0.8 ± 0.2 g/L **Apo-B/Apo-A1** With DR: 0.7 ± 0.2 Without DR: 0.6 ± 0.2	**TG** With DR: 1.3 (0.9, 1.9) mmol/L Without DR: 1.3 (0.9, 1.9) mmol/L **HDL** With DR: 1.3 ± 0.5 mmol/L Without DR: 1.5 ± 0.4 mmol/L **LDL** With DR: 2.5 ± 0.9 mmol/L Without DR: 2.5 ± 0.7 mmol/L	**Odds ratio (95% CI) for any DR per SD increase of**: Apo-A1: 0.76 (0.59–0.98) Apo-B: 1.31 (1.02–1.68) Apo-B/Apo-A1: 1.48 (1.13–1.95) **Odds ratio (95% CI) for VTDR per SD increase of**: Apo-A1: 0.53 (0.38–0.76) Apo-B: 1.47 (1.10–1.96) Apo-B/Apo-A1: 1.76 (1.27–2.45)
Malaguarnera et al. [19] (2013)	With DR: 67 Without DR: 78	**Apo-A1** With DR: 1.54 ± 0.18 g/L Without DR: 1.47 ± 0.20 g/L **Apo-B** With DR: 1.08 ± 0.21 g/L Without DR: 1.06 ± 0.19 g/L **Lp(a)** With DR: 56.4 ± 28.2 mg/dL Without DR: 34.1 ± 12.4 mg/dL	**TG** With DR: 1.67 ± 0.76 mmol/L Without DR: 1.48 ± 0.88 mmol/L **HDL** With DR: 1.44 ± 0.36 mmol/L Without DR: 1.36 ± 0.30 mmol/L **LDL** With DR: 4.19 ± 0.99 mmol/L Without DR: 3.87 ± 0.95 mmol/L	**Mean difference between groups for each parameter**: Apo-A1: *p* = 0.03 Apo-B: *p* = 0.55 Lp(a): *p* < 0.01 LDL: *p* = 0.05 HDL: *p* = 0.15 TG: *p* = 0.17
Aryan et al. [21] (2017)	With DR: 444 Without DR: 439	**Apo-A1** With DR: 141.8 ± 28.5 mg/dL Without DR: 135.6 ± 27.9 mg/dL **Apo-B** With DR: 88.1 ± 27.5 mg/dL Without DR: 85.8 ± 26.2 mg/dL **Lp(a)** With DR: 35.9 ± 42.2 mg/dL Without DR: 31.5 ± 34.4 mg/dL **Apo-B/Apo-A1** With DR: 0.63 ± 0.24 Without DR: 0.64 ± 0.24	**TG** With DR: 175.9 ± 82.9 mg/dL Without DR: 170.8 ± 111.0 mg/dL **HDL** With DR: 45.7 ± 11.8 mg/dL Without DR: 45.6 ± 12.4 mg/dL **LDL** With DR: 106.5 ± 34.6 mg/dL Without DR: 102.5 ± 31.4 mg/dL	**Logistic regression analysis for association with DR**: Lp(a): β 0.37; OR 2.8; 95% CI 0.93–8.4 (*p* = 0.37) Apo-A1: β 0.01; OR 1.1; 95% CI 0.41–2.9 (*p* = 0.34) Apo-B: β 0.20; OR 2.4; 95% CI 0.8–7.2 (*p* = 0.87) Apo-B/Apo-A1: β 0.56; OR 3.4; 95% CI 0.89–12.9 (*p* = 0.45)
Zhang et al. [25] (2018)	With DR: 315 Without DR: 708	**Apo-A1** With DR: 7.3 ± 1.2 μmol/L Without DR: 7.5 ± 1.2 μmol/L **Apo-CIII** With DR: 6.4 ± 1.1 μmol/L Without DR: 6.2 ± 1.1 μmol/L **Apo-D** With DR: 3.9 ± 0.6 μmol/L Without DR: 4.0 ± 0.6 μmol/L **Apo-E** With DR: 1.2 ± 0.2 μmol/L Without DR: 1.1 ± 0.2 μmol/L	**TG** With DR: 1.3 ± 0.4 mmol/L Without DR: 1.2 ± 0.5 mmol/L **HDL** With DR: 1.4 ± 0.6 mmol/L Without DR: 1.5 ± 0.5 mmol/L	**Cox proportional hazard analysis for DR risk**: Apo-A1 ≥ 7.4: HR 0.86; 95% CI 0.70–0.99 Apo-CIII ≥ 6.3: HR 1.25; 95% CI 1.04–1.49 Apo-E ≥ 1.1: HR 1.23; 95% CI 1.03–1.47 Apo-CIII/Apo-A1 ≥ 0.9: HR 1.34; 95% CI 1.11–1.60 Apo-E/Apo-A1 ≥ 0.2: HR 1.21; 95% CI 1.01–1.46
Chung et al. [26] (2019)	Without DR: 743 With DR: 472	**Apo-A1** With DR: 1.20 (1.17–1.23) g/L Without DR: 1.26 (1.24–1.29) g/L **Apo-B** With DR: 0.86 (0.84–0.89) g/L Without DR: 0.87 (0.85–0.90) g/L **Apo-B/Apo-A1** With DR: 0.72 (0.70–0.74) Without DR: 0.75 (0.73–0.78)	**TG** With DR: 1.46 (1.39–1.53) mmol/L Without DR: 1.54 (1.48–1.60) mmol/L **HDL** With DR: 1.12 ± 0.37 mmol/L Without DR: 1.17 ± 0.35 mmol/L **LDL** With DR: 2.78 ± 1.01 mmol/L Without DR: 2.84 ± 1.03 mmol/L	**Logistic regression analyses for DR risk**: Apo-A1 OR per SD increase 0.55; 95% CI: 0.32–0.97 (*p* = 0.04) Apo-B OR per SD increase 1.28; 95% CI: 0.85–1.93 (*p* = 0.23) Apo-B/Apo-A1 OR per SD increase 2.83; 95% CI: 1.18–6.76 (*p* = 0.02)
Liu et al. [29] (2022)	With DR: 309 Without DR: 186	**Apo-A1** With DR: 1.29 ± 0.29 mmol/L Without DR: 1.34 ± 0.41 mmol/L **Apo-B** With DR: 1.01 ± 0.34 mmol/L Without DR: 1.02 ± 0.26 mmol/L **Apo-E** With DR: 45.74 ± 20.80 mmol/L Without DR: 47.36 ± 24.96 mmol/L **Lp(a)** With DR: 179.12 ± 256.68 mmol/L Without DR: 141.64 ± 183.54 mmol/L	**TG** With DR: 1.80 ± 1.26 mmol/L Without DR: 2.24 ± 2.01 mmol/L **HDL** With DR: 1.25 ± 0.34 mmol/L Without DR: 1.40 ± 2.13 mmol/L **LDL** With DR: 2.85 ± 1.11 mmol/L Without DR: 3.00 ± 0.88 mmol/L	**Logistic regression analyses for DR risk**: Apo-A1 OR 1.91; 95% CI: 0.94–3.88 (*p* = 0.07) Apo-B OR 7.04; 95% CI: 3.37–14.70 (*p <* 0.001) Apo-E OR 1.06; 95% CI: 1.04–1.08 (*p* < 0.001) Lp(a) OR 1.00; 95% CI: 1.00–1.00 (*p =* 0.27)

Abbreviations: DR: diabetic retinopathy; HR: hazard ratio; OR: odds ratio; SD: standard deviation; CI: confidence interval; apo: apolipoprotein; Lp(a): lipoprotein (a); TG: triglyceride; HDL: high density lipoprotein; LDL: low density lipoprotein.

**Table 3:** Summary of studies on the association of apolipoproteins and cholesterol parameters with DR severity.

Author	Number of Participants	Mean Levels of Apolipoprotein Parameters	Mean Levels of Cholesterol Parameters	Outcomes Related to DR Severity
Deguchi et al. [17] (2011)	NPDR: 34 PDR: 82	**Apo-A1** NPDR: 145.6 ± 21.2 mg/dL PDR: 136.9 ± 24.9 mg/dL **Apo-B** NPDR: 90.8 ± 21.4 mg/dL PDR: 102.5 ± 25.3 mg/dL **Apo-B/Apo-A1** NPDR: 0.64 ± 0.20 PDR: 0.77 ± 0.24	**TG** NPDR: 121.3 ± 52.4 mg/dL PDR: 139.1 ± 92.9 mg/dL **HDL** NPDR:55.4 ± 12.3 mg/dL PDR: 47.8 ± 13.0 mg/dL **LDL** NPDR: 108.8 ± 32.0 mg/dL PDR: 118.9 ± 33.5 mg/dL	**Apo-B/Apo-A1 to PDR**: Standard regression coefficient 0.28 t value 3.06 *p* = 0.003
Hu et al. [18] (2012)	Mild NPDR: 25 PDR: 25	**Apo-A1** Mild NPDR: 1.38 ± 0.22 g/L PDR: 1.22 ± 0.26 g/L **Apo-B** Mild NPDR: 0.78 ± 0.16 g/L PDR: 0.82 ± 0.18 g/L **Apo-E** Mild NPDR: 43.02 ± 11.12 mg/L PDR: 43.57 ± 13.10 mg/L **Apo-A1/Apo-B** Mild NPDR: 1.81 ± 0.40 PDR: 1.52 ± 0.33	**TG** Mild NPDR: 1.82 ± 0.85 mmol/L PDR: 1.97 ± 0.86 mmol/L **HDL** Mild NPDR: 1.36 ± 0.30 mmol/L PDR: 1.19 ± 0.27 mmol/L **LDL** Mild NPDR: 3.30 ± 1.12 mmol/L PDR: 3.41 ± 1.08 mmol/L	**Logistic regression analysis for association with PDR**: Apo-A1: OR 0.026; 95% CI < 0.01–0.45 (*p* = 0.03) Apo-A1/Apo-B: OR 0.05, 95% CI 0.01–0.42 (*p* = 0.02)
Crosby-Nwaobi et al. [13] (2015)	NPDR: 252 PDR: 128	**Apo-A** NPDR: 1.4 ± 0.5 g/L PDR: 1.5 ± 0.3 g/L **Apo-B** NPDR: 0.5 ± 0.5 g/L PDR: 0.8 ± 0.2 g/L **Apo-B/Apo-A** NPDR: 0.39 ± 0.32 PDR: 0.54 ± 0.18	Not reported	**Multinomial logistic regression analysis for (compared with NPDR without CSME as reference)**: PDR (without CSME): Apo-B: OR 1.20; 95% CI 1.06–1.36 (*p* <0.01) Apo-B/Apo-A: OR 1.18; 95% CI 1.01–1.38 (*p* = 0.04) PDR (with CSME): Apo-B: OR 1.20; 95% CI 1.01–1.43 (*p* = 0.04) Apo-B/Apo-A: OR 1.25; 95% CI 0.99–1.59 (*p* = 0.06)
Prakash et al. [20] (2016)	Mild NPDR: 4 Moderate NPDR: 8 VTDR:12	**Apo-A1** * Mild NPDR: 211.16 ± 2.02 Moderate NPDR: 168.77 ± 33.97 VTDR: 132.25 ± 2.02 **Apo-B** * Mild NPDR: 99.40 ± 0.69 Moderate NPDR: 144.50 ± 36.33 VTDR: 188.98 ± 41.57 **Apo-B/Apo-A1** Mild NPDR: 0.67 ± 0.08 Moderate NPDR: 1.11 ± 0.28 VTDR: 1.66 ± 0.56	**TG** * Mild NPDR: 130 ± 0 Moderate NPDR: 136 ± 16.47 VTDR: 163.08 ± 23.06 **HDL** * Mild NPDR: 45.50 ± 4.0 Moderate NPDR: 47.75 ± 6.6 VTDR: 40 ± 6.0 LDL* Mild NPDR: 119.90 ± 13.97 Moderate NPDR: 119.90 ± 28.59 VTDR: 139.83 ± 19.78	**Mean difference between groups for each parameter**: Apo-A1: *p* < 0.01 Apo-B: *p* < 0.01 Apo-B/Apo-A1: *p* < 0.01 LDL: *p* = 0.10 HDL: *p* = 0.02 TG: *p* = 0.01
Sharma et al. [22] (2017)	NPDR: 49 PDR: 48	**Apo-A1** NPDR: 126.9 ± 12.5 mg/dL PDR: 118.6 ± 9.7 mg/dL **Apo-B** NPDR: 94.5 ± 17.7 mg/dL PDR: 101.5 ± 15.7 mg/dL **Apo-B/Apo-A1** NPDR: 0.7 ± 0.1 PDR: 0.8 ± 0.1	**TG** NPDR: 145.7 ± 60.0 mg/dL PDR: 165.0 ± 63.3 mg/dL **HDL** NPDR: 48.1 ± 14.7 mg/dL PDR: 45.9 ± 10.9 mg/dL **LDL** NPDR: 108.5 ± 37.5 mg/dL PDR: 121.2 ± 41.4 mg/dL	**Mean difference between groups**: Apo-A1: *p* < 0.01 Apo-B: *p* < 0.01 Apo-B/Apo-A1: *p* < 0.01 TG: *p* < 0.05 HDL: *p* = 0.14 LDL: *p* < 0.05
Mukherjee et al. [23] (2017)	Mild NPDR: 25 Mod NPDR: 25 Severe NPDR: 25 PDR: 35	**Apo-A1** Mild NPDR: 1.47 ± 0.12 mg/dLMod NPDR: 1.23 ± 0.21 mg/dL Severe NPDR: 1.67 ± 0.17 mg/dL PDR: 1.21 ± 0.34 mg/dL **Apo-B** Mild NPDR: 0.84 ± 0.08 mg/dL Mod NPDR: 0.67 ± 0.13 mg/dL Severe NPDR: 1.14 ± 0.18 mg/dL PDR: 1.13 ± 0.43 mg/dL	**TG** Mild NPDR: 132.3 ± 27.6 mg/dL Mod NPDR: 167.7 ± 28.7 mg/dL Sev NPDR: 286.4 ± 64.4 mg/dL PDR: 191.1 ± 50.8 mg/dL **HDL** Mild NPDR: 41.0 ± 3.6 mg/dL Mod NPDR: 36.6 ± 2.5 mg/dL Severe NPDR: 39.8 ± 3.2 mg/dL PDR: 41.6 ± 3.4 mg/dL **LDL** Mild NPDR: 116.2 ± 31.9 mg/dL Mod NPDR: 113.8 ± 23.8 mg/dL Sev NPDR: 183.8 ± 69.8 mg/dL PDR: 99.8 ± 38.4 mg/dL	**Significant mean difference (*p* < 0.05) between groups for each parameter**: Apo-A1: Between any type of DR with patients without DR. Apo-B: Between severe NPDR and PDR with patients without DR.
Ankit et al. [24] (2017)	Mild NPDR: 50 Moderate NPDR: 38 Severe NPDR: 20 PDR: 9	**Apo-A1** Mild NPDR: 148.86 mg/dL Moderate NPDR: 124.39 mg/dL Severe NPDR: 110.30 mg/dL PDR: 103.22 mg/dL **Apo-B** Mild NPDR: 74.98 mg/dL Moderate NPDR: 96.45 mg/dL Severe NPDR: 101.00 mg/dL PDR: 108.11 mg/dL **Apo-B/Apo-A1** Mild NPDR: 0.51 Moderate NPDR: 0.78 Severe NPDR: 0.92 PDR: 1.05	**HDL** Mild NPDR: 39.96 mg/dL Moderate NPDR: 36.74 mg/dL Severe NPDR: 32.95 mg/dL PDR: 29.33 mg/dL **LDL** Mild NPDR: 119.74 mg/dL Moderate NPDR: 124.76 mg/dL Severe NPDR: 132.60 mg/dL PDR: 128.11 mg/dL **TG** Not reported	Apo-B/Apo-A1 had a stronger correlation with DR severity (*p* ≤ 0.001) compared with Apo-B or Apo-A1 alone.
Moosaie et al. [27] (2020)	NPDR: 162 PDR: 163	**Apo-A1** NPDR: 129.8 ± 25.3 g/L PDR: 128 ± 28.85 g/L **Apo-B** NPDR: 92.2 ± 25.8 g/L PDR: 98.2 ± 27.1 g/L Apo-A1/Apo-B NPDR: 0.8 ± 0.4 PDR: 0.76 ± 0.31 **Lp(a)** NPDR: 65.8 ± 28.0 mg/dL PDR: 63.81 ± 35.97 mg/dL	**TG** NPDR: 160.7 ± 80.8 mg/dL PDR: 168.26 ± 110.76 mg/dL **HDL** NPDR: 44.7 ± 10.8 mg/dL PDR: 45.69 ± 11.41 mg/dL **LDL** NPDR: 100.9 ± 32.4 mg/dL PDR: 100.87 ± 31.95 mg/dL	**Logistic regression analysis for the association with DR severity**: Lp(a): OR 1.00; 95% CI 0.1–1.01 (*p* = 0.29) Apo-A1: OR 0.42; 95% CI 0.14–1.3 (*p* = 0.13) Apo-B: OR 1.02; 95% CI 1.01–1.04 (*p* < 0.01) Apo-B/Apo-A1: OR 1.07; 95% CI 1.03–1.1 (*p* < 0.01)
Hanifah et al. [28] (2021)	Sev NPDR: 6 Early PDR: 9 High risk PDR: 20 Advanced PDR: 11	**Apo-A1** Sev NPDR: 1.4 ± 0.2 mg/dL Early PDR: 1.5 ± 0.2 mg/dL High risk PDR: 1.4 ± 0.2 mg/dL Advanced PDR: 1.5 ± 0.2 mg/dL **Apo-B** Sev NPDR: 0.9 ± 0.2 mg/dL Early PDR: 1.2 ± 0.2 mg/dL High risk PDR: 1.3 ± 0.3 mg/dL Advanced PDR: 1.1 ± 0.3 mg/dL **Apo-B/Apo-A1** Sev NPDR: 0.7 ± 1.0 Early PDR: 0.8 ± 0.2 High risk PDR: 0.9 ± 0.2 Advanced PDR: 0.8 ± 0.3	Not reported	**Mean difference between groups**: Apo-A1: *p* = 0.32 Apo-B: *p* = 0.06 Apo-B/Apo-A1: *p* = 0.08

* Unit of measurement not reported; Abbreviations: NPDR: nonproliferative diabetic retinopathy; PDR: proliferative diabetic retinopathy; VTDR: vision-threatening diabetic retinopathy; CSME: clinically significant macular edema; OR: odds ratio; CI: confidence interval; apo: apolipoprotein; Lp(a): lipoprotein (a); TG: triglyceride; HDL: high density lipoprotein; LDL: low density lipoprotein.

## Data Availability

Not applicable.
